# Estimating the burden of adult hospital admissions due to RSV and other respiratory pathogens in England

**DOI:** 10.1111/irv.12910

**Published:** 2021-10-17

**Authors:** Ashley Sharp, Mehdi Minaji, Nikolaos Panagiotopoulos, Rachel Reeves, Andre Charlett, Richard Pebody

**Affiliations:** ^1^ Field Epidemiology Training Programme Public Health England London UK; ^2^ Public Health England London UK

**Keywords:** burden of disease, hospital admission, respiratory, respiratory syncytial virus, time series analysis

## Abstract

Respiratory syncytial virus (RSV) is a common seasonal respiratory virus and an important cause of illness among infants, but the burden of RSV disease is not well described among the older population. The objective of this study was to estimate the age‐specific incidence of hospital admission among over 65 s due to respiratory illnesses attributable to RSV in England to inform optimal vaccine and therapeutic interventions.

We used linear multiple regression to examine the effect of changes in weekly counts of respiratory pathogens on the weekly counts of respiratory hospital admissions. The study population was all patients aged 65 years or over admitted to English hospitals between 2nd August 2010 and 30th July 2017.

RSV was estimated to account for a seasonal annual average of 71 (95% CI 52–90) respiratory admissions per 100 000 in adults age 65–74 and 251 (95% CI 186–316) admissions per 100,000 adults age 75+. Pneumococcus was the pathogen responsible for highest annual average respiratory admission with 448 (95% CI 310–587) admissions per 100,000 adults age 65–74 and 1010 (95% CI 527–1493) admissions per 100,000 adults aged 75+.

This study shows that RSV continues to exert a significant burden of disease among older adults in England. These findings will support development of policy for the use of RSV therapeutics and vaccines in this age group.

## 1 INTRODUCTION

Respiratory syncytial virus (RSV) is a common seasonal respiratory virus and is recognized as an important cause of illness among infants but also increasingly amongst adults.^1^, ^2^, ^3^, ^4^, ^5^, ^6^, ^7^, ^8^, ^9^ Understanding the impact of RSV on morbidity, mortality, and healthcare usage is important to inform public health policy. However, the burden of RSV disease is not well described among the older population.^10^ RSV infection causes a similar clinical syndrome to many other viruses, including influenza; in this age group,^11^ there is little routine diagnostic testing for respiratory viruses in many countries including England, and finally patients may present late with complications of infection when the virus is no longer detectable. There are many candidate RSV vaccines and therapeutics in advanced development.^12^, ^13^, ^14^, ^15^ Without a clear picture of the impact of RSV, we cannot fully appraise these options.

Existing surveillance data in England show seasonal peaks in RSV infection occurring with regularity usually around November, accompanied by autumn–winter peaks in other respiratory viruses such as influenza. Time series modelling offers tools to understand the role of seasonal respiratory viruses in driving hospital admissions and excess winter deaths due to respiratory disease. Multivariable regression analysis comparing the weekly variation in outcomes such as hospital admissions or primary care consultations, with variation in explanatory factors such as laboratory diagnoses of pathogens, allows us to model the proportion that could be attributable to each factor. Using routine healthcare and laboratory data from national administrative data systems, this approach has been used to measure the burden of seasonal infections such as rotavirus,^16^ influenza,^17^, ^18^ RSV,^19^, ^20^, ^21^ and other respiratory pathogens.^22^, ^23^ Fleming et al.^19^ used influenza and RSV laboratory data between 1995 and 2009 and estimated that in elderly patients, RSV in England was comparable to influenza in terms of the burden of hospital admissions and deaths. Reeves et al.^20^ used data on 10 different respiratory pathogens to model the cause‐specific burden of RSV disease in under 5‐year‐olds also in England.

### 1.1 Objective

The objective of this study was to estimate the age‐specific incidence of hospital admission among over 65 s due to respiratory illnesses attributable to RSV, in order to inform future intervention policies.

## 2 METHODS

We used linear multiple regression to examine the effect of changes in weekly counts of respiratory pathogens (independent variables) on the weekly counts of respiratory hospital admissions (dependent variable). The study population was all patients aged 65 years or over admitted to English hospitals between 2nd August 2010 and 30th July 2017.

UK laboratory data, including diagnoses of viral infections, is compiled using the Second‐Generation Surveillance System (SGSS). SGSS contains all positive results from diagnostic tests conducted in Public Health England (PHE), National Health Service (NHS), and private microbiology laboratories in England and Wales since 1990. Data collected includes specimen information, test type, patient identifiable information, and laboratory information. To account for repeated testing, identical results within 14 days of each other are grouped as a single episode of infection and assigned a unique organism‐patient‐illness‐episode identifier (OPIE ID). The coverage of SGSS is very high. PHE does not collect data on the ascertainment of RSV tests in SGSS, but studies considering the ascertainment of other clinically significant infections (e.g., blood‐stream infections, methicillin‐resistant *Staphylococcus aureus*) estimate ascertainment to be approximately 70%–90% and consistent all year round.

Hospital data in England are collected in the Hospital Episode Statistics (HES) database. The HES Admitted Patient Care Database contains information for all patients admitted to all NHS hospitals in England, and all patients treated in private hospitals paid for by the NHS. Data collected includes: clinical information including diagnoses (ICD‐10) and procedures, patient identifiable information, geographical information and administrative information including admission and discharge dates. Each entry in HES relates to an episode of care under a single consultant doctor. A “spell” is defined as the time between the admission and discharge episodes within a hospital, while a “continuous inpatient spell” (CIP) is the time from admission to final discharge, allowing for transfers between hospitals. We have seen a steady increase in healthcare usage in England, related to the ageing population and greater access to healthcare.

### 2.1 Laboratory data collection

For the laboratory data (SGSS), we selected all records of respiratory pathogens from English laboratories with a specimen date from 2nd August 2010 to 30th July 2017, including all age groups. The list of respiratory pathogens is presented in Table 1, along with the specimen sites and test methods included. We excluded records with the following recorded test methods: antibody detection, electron microscopy, light microscopy, and “unknown.” We checked records for specimen dates and age fields and removed records where this information was missing. We included only the first record for each OPIE ID. We then created tables of weekly counts of each pathogen and visually examined each time series.

**TABLE 1:** Pathogens, specimen sites, and test methods included

	Pathogen	
Viruses	RSV, influenza A, influenza B, rhinovirus, parainfluenza, human metapneumovirus (hMPV), adenovirus, coxsackievirus, rhinovirus, echovirus, enterovirus	
Bacteria	*Streptococcus pneumoniae, Haemophilus influenzae, Mycoplasma pneumoniae*, *Klebsiella pneumoniae*, *Pseudomonas aeruginosa*, *Staphylococcus aureus*, *Streptococcus* Group A, *Streptococcus* Group B, *Escherichia coli*, *Chlamydia trachomatis*, *Bordetella pertussis, Legionella pneumophila*	

	Specimen sites	Pathogen
Upper respiratory	Nose, throat, nasopharyngeal aspirate, per‐nasal, pharyngeal, upper respiratory tract	Viruses & pertussis
Lower respiratory	Alveolar lavage, bronchial, endotracheal, lower respiratory tract, lung, sputum, trachea, chest drain, pleural	All pathogens
Normally sterile site	Ascitic fluid, blood, cerebrospinal fluid	Bacteria (excluding pertussis) & enterovirus

	Test method	
	Antigen detection, culture, and genomic/PCR/LCR detection	

### 2.2 Hospital data collection

For the hospital admissions data (HES), we considered one admission as one continuous inpatient spell (CIP). We selected admissions to English hospitals among patients aged 65 or older, with an admission date from 2nd August 2010 to 30th July 2017, with a primary diagnosis in the first episode of the first spell of a cardio‐respiratory disease (I00‐J99) or urinary tract infection (N39) (control). Only the primary diagnosis of the first episode of the spell was considered to avoid double counting of admissions that had two or more respiratory diagnoses. We cleaned the hospital data, examining the admission dates and age at start of admission and removing any entries where this information was missing. We also examined the admission method and removed any non‐emergency admissions. We counted admissions per week for each of the outcomes in Table 2. We then created tables of weekly counts of each outcome by age group (65–74 and 75+) and visually examined each time series.

**TABLE 2:** Outcome groups and ICD 10 codes, demonstrating hierarchy

Outcome	ICD 10 codes
• Cardiorespiratory disease	I00–J99
• Respiratory disease	J00–J99
• Influenza and pneumonia	J09–J18
• Bronchitis, bronchiolitis and unspecified LRTI	J20–J22, J40
• Chronic respiratory disease	J41–J47
• Urinary tract infection (control)	N39

### 2.3 Data analysis

We included RSV in all models along with three “time variables”: (1) a secular trend (to account for an overall increase in admissions over the seven‐year study period), (2) a 1‐year period term (to account for unmeasured seasonal factors that contribute to hospital admissions), and (3) a categorical season term (to account for any step changes in testing practice and influenza activity by season), all three of which we found to improve the models. Then, we used a backward stepwise approach, starting with all pathogens then removing first those pathogens with negative coefficients (starting with the most negative) due to biological implausibility, then those with positive coefficients that did not contribute significantly to the model (starting with the coefficient with the largest *p* value). We assessed the effect of a lag of up to 4 weeks, first for RSV, then for the other remaining pathogens (starting with the coefficient with the smallest *p* value), checking the effect on each model and selecting the overall best lag for each pathogen to apply to all models. We explored the interaction between RSV and influenza A with pneumococcus and the removal of pneumococcus. We did not split influenza A into H1 and H3 subtypes as subtype data was not available. We assessed model fit using the Akaike information criterion (AIC) and the coefficient of determination (*R* ^2^). We fitted models to the control data using the same three “time variables” and same lag as other models.

We interpreted the coefficients for each pathogen as the number of admissions attributable to each case of infection. We calculated the number of weekly admissions attributable to each pathogen by multiplying the number of cases of each infection in the laboratory database by the coefficient for that pathogen, so for RSV in week n (RSV_n_), where *β*_1_ is the model coefficient for RSV, the RSV attibutable admissions in week n is given by:

$$\mathrm{RSV}\;{\text{attributable admissions}}_{n}=\beta_{1}{\mathrm{RSV}}_{n}.$$

We calculated the upper and lower 95% confidence intervals by multiplying the number of cases by the upper and lower confidence interval estimates of the coefficients. We calculated the overall and seasonal number of admissions attributable to each pathogen by taking the sum of weekly admissions. We calculated the seasonal rate of admissions based on the average Office for National Statistics mid‐year population estimates for the 2 years either side of the season. We plotted time series showing the actual and expected weekly admissions, and the weekly admissions attributable to each pathogen. We conducted the analysis using R version 4.0.2.

### 2.4 Ethical statement

SGSS is held by PHE under existing information governance policies. HES is accessed by PHE under existing information governance policies. All data is anonymized.

## 3 RESULTS

We identified 4,254,927 hospital admissions with a cardio‐respiratory diagnosis (I00‐J99) for inclusion in the study and 1,092,778 laboratory records including 39,219 cases of RSV.

**FIGURE 1:**
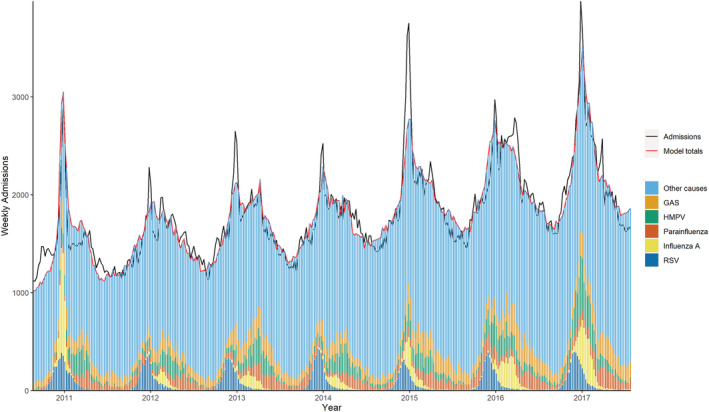
Weekly observed and predicted respiratory (J00–J99) admissions attributable to each pathogen, age 65–74

**FIGURE 2:**
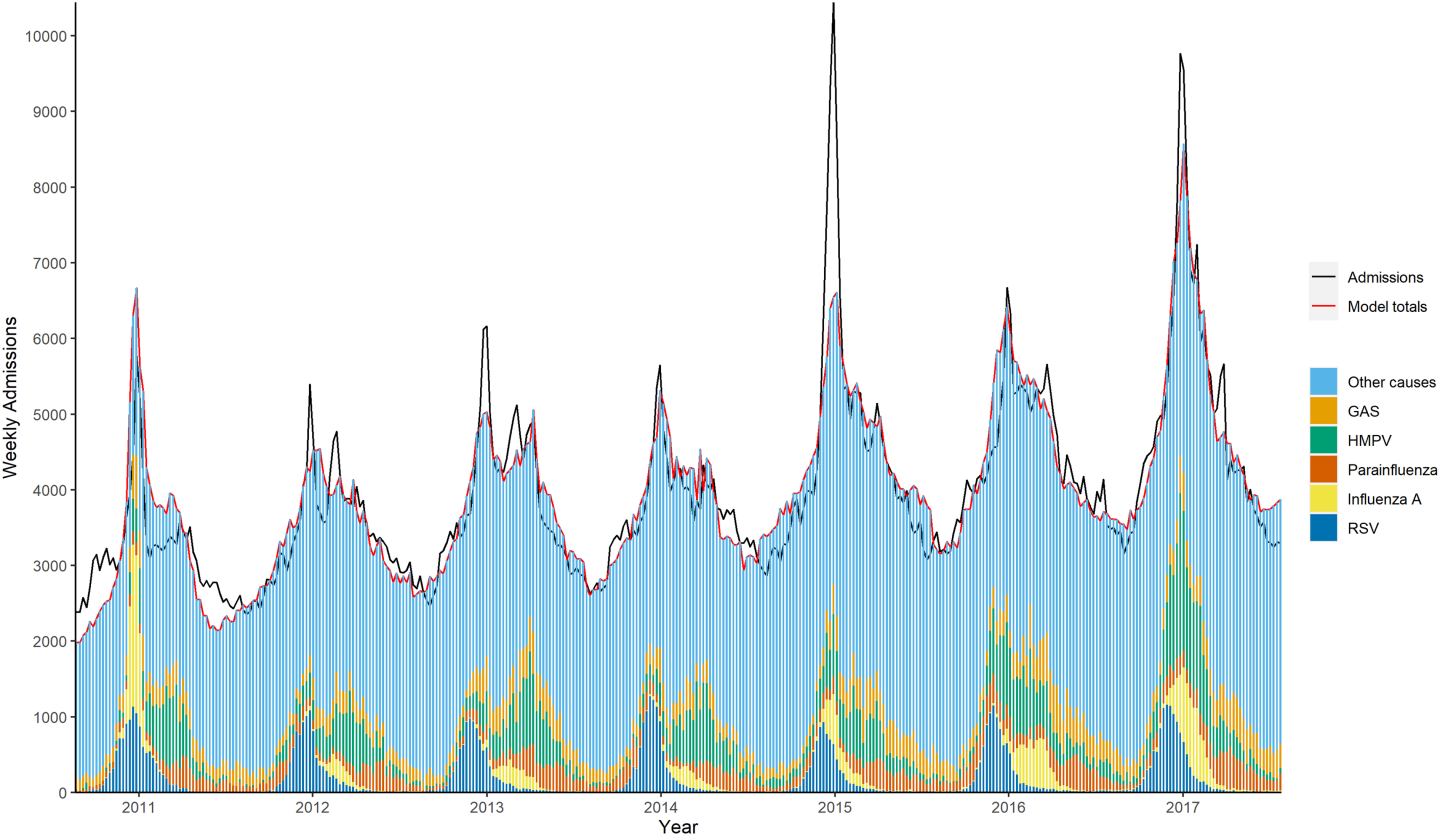
Weekly observed and predicted respiratory (J00–J99) admissions attributable to each pathogen, age 75+

All 10 models included a secular trend, a one‐year period term, a categorical season term and weekly counts of RSV, influenza A, HMPV, and pneumococcus. Five models also included parainfluenza and three included group A streptococcus. We found model fit improved overall with a lag of 3 weeks for RSV, and 1 week for HMPV. Adding an interaction between RSV and pneumococcus led to a negative coefficient for RSV, and adding an interaction between influenza A and pneumococcus did not improve the model fit. Removal of pneumococcus gave a poorer model fit and did not significantly change the respiratory admission rates for the other pathogens. Figures 1 and 2 below show model fit for two models: respiratory admissions (J00–J99) in 65–74 years and in 75+ years, with the weekly admissions predicted by the model and the weekly proportion attributable to each pathogen predicted by the model, along with the actual admissions.

Table 3 shows data from the same two models (respiratory admissions (J00–J99) in 65–74 years and in 75+ years) with the mean seasonal rate of admissions attributable to each pathogen. The highest rate of admissions in both age groups was attributable to pneumococcus, with a seasonal average of 448 (310–587) admissions per 100 000 adults age 65–74 and 1010 (527–1493) admissions per 100 000 adults aged 75+. RSV was estimated to account for a seasonal average of 71 (52–90) respiratory admissions per 100 000 adults age 65–74 and 251 (186–316) admissions per 100 000 adults age 75+. The mean seasonal rate of admissions for RSV was significantly higher than influenza A for adults age 75+.

**TABLE 3:** Mean seasonal respiratory (J00–J99) admissions attributable to each pathogen by age group

Organism	Age group	Admissions(mean)	Admissions(95 CI)	Rate/100,000(mean)	Rate(95% CI)
Pneumococcus	65–74	22 610	15 626–29 595	448	310–587
	75+	43 641	22 779–64 503	1010	527–1493
HMPV	65–74	5023	3554–6492	99	70–128
	75+	16 781	12 387–21 174	387	286–488
GAS	65–74	4815	912–8718	95	18–172
	75+	‐	‐	‐	‐
RSV	65–74	3565	2632–4498	71	52–90
	75+	10 808	7997–13 620	251	186–316
Parainfluenza	65–74	2540	358–4722	50	7–93
	75+	6784	142–13 427	157	3–310
Influenza A	65–74	2273	1721–2824	45	34–56
	75+	5141	3564–6718	119	82–155

**FIGURE 3:**
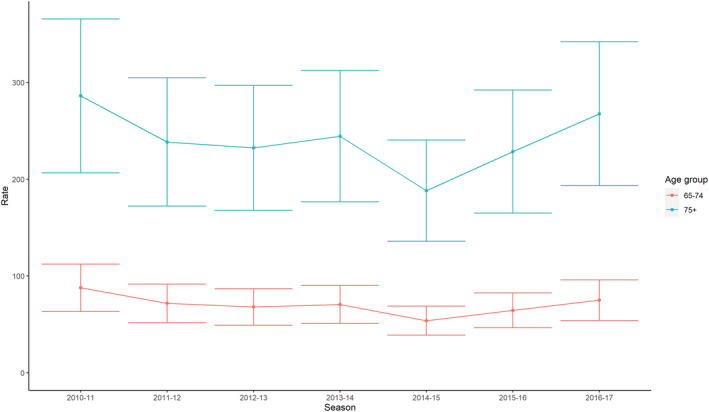
RSV attributable respiratory (J00–J99) admission rate by season and age group, 95% CI

Figure 3 shows time series data from the same two models (respiratory admissions (J00–J99) in 65–74 years and in 75+ years), showing the seasonal trend in the RSV attributable respiratory (J00–J99) admission rate attributable by age group. There was no significant change in admission rate over the study period.

Table 4 combines data from all 10 models with the mean seasonal RSV attributable admissions for each outcome by age group and diagnostic code. For each outcome, the admission rate was higher in the 75+ age group.

**TABLE 4:** Mean seasonal RSV attributable admissions by diagnosis and age group, England, 2010–2017

Admission group	Age group	Admissions(mean)	Admissions(95% CI)	Rate/100,000(mean)	Rate/100,000(95% CI)
Cardiorespiratory disease I00–J99	65–74	3062	2093–4032	61	42–80
	75+	9978	6989–12 967	232	162–301
Respiratory disease J00–J99	65–74	3565	2632–4498	71	52–90
	75+	10 808	7997–13 620	251	186–316
Influenza and Pneumonia J09–J18	65–74	1263	874–1653	25	17–33
	75+	5073	3621–6524	118	84–151
Bronchitis J20–J22, J40	65–74	599	417–781	12	8–16
	75+	2506	1828–3183	58	42–74
Chronic respiratory disease J41–J47	65–74	1569	1174–1964	31	23–39
	75+	2711	2085–3336	63	48–77
Urinary disease	65–74	‐	‐	‐	‐
	75+	510	11–1009	12	0–23

## 4 DISCUSSION

This study found that RSV disease among adults in England aged 65–74 and 75+ accounted for an average annual hospital admission rate of 71 and 251 per 100 000 respectively. The yearly burden remained stable over the seven‐year study period from 2010 to 2017. The RSV burden was greater than influenza A and parainfluenza, similar to HMPV and GAS and greatly exceeded by pneumococcus. Among the specific respiratory subdiagnoses, influenza and pneumonia were the most common RSV attributable admission, followed by bronchitis and chronic respiratory disease. This study used nationally representative data sets and examined the full range of known pathogens likely to account for seasonal respiratory admissions.

Comparing these results with Fleming's analysis,^19^ we find no statistically significant differences, noting the wide confidence intervals in both studies, though point estimates did vary somewhat. This study found a lower rate of respiratory disease in the 65–74 age group at 71 (52–90) compared to Fleming's 86 (62–101), and a slightly higher rate in the 75+ group at 251 (186–316)

Although the primary objective of the study was to assess RSV burden, the methodology does provide evidence of the impact of other pathogens in this age‐group notably pneumococcus, HMPV, GAS and parainfluenza. There are limitations inherent in this study methodology. The observation that RSV explained some urinary disease in the over 75's, albeit with very wide confidence intervals, despite the observation that urinary disease has no particular seasonality, indicates the limitations of this methodology, and also points to the complexity of causal factors contributing to respiratory admissions in the elderly. The model makes the reasonable assumption of a linear, additive relationship between different pathogens. We may argue whether a proportion of the pneumococcus admissions are secondary to RSV or other viruses and therefore potentially partially preventable with future RSV intervention strategies. Only invasive isolates of pneumococcus were included so the burden estimate is likely correct. The laboratory testing data includes all age groups and settings. We were unable to distinguish between tests done in hospital and the community, and most of the testing was done in children, which may explain the lagged peak in admissions in the elderly as reflecting a sequential pattern of infection across the age groups perhaps combined with delayed onset of complications such as secondary bacterial infection. We did not split influenza A into H1 and H3 subtypes but experience from Cromer et al. suggested this would not improve the model.^17^ We did not include smoothing as is this is primarily a descriptive technique. The model retains periodic terms to account for unmeasured seasonal factors. Future studies could perhaps be strengthened by combining infectious disease data with environmental data such as air quality and temperature. A recent study^24^ used linked data on positive and negative RSV test results and hospital admissions to develop a predictive model of RSV attributable hospital admissions in under 5's, which gave an overall very similar estimate of disease burden as with linear regression among the same population. A similar approach could be used to study disease burden in the elderly.

### 4.1 Conclusion and recommendations

This study shows that RSV continues to exert a significant burden of disease among older adults in England. This confirms the importance of RSV as a cause of hospital admissions among the elderly. There is also evidence of an on‐going burden due to pneumococcus and potentially important burden of HMPV in this age group. There is more work required using a range of analytical approaches including data linkage. These findings will support development of policy for the use of RSV therapeutics and vaccines in this age group, as these products become available and licensed for use.

## CONFLICT OF INTEREST

The authors declare no conflicts of interest.

## DISCLAIMER

The views expressed in this article are those of the authors and are not necessarily those of Public Health England or the Department of Health and Social Care.

## AUTHOR CONTRIBUTIONS

**Ashley Sharp:** Data curation; formal analysis; investigation; methodology; visualization. **Mehdi Minaji:** Formal analysis; investigation; methodology. **Nikolaos Panagiotopoulos:** Formal analysis; investigation; methodology. **Rachel Reeves:** Conceptualization; methodology; supervision. **Andre Charlett:** Formal analysis; methodology; supervision. **Richard Pebody:** Conceptualization; formal analysis; investigation; methodology; supervision.

### PEER REVIEW

The peer review history for this article is available at https://publons.com/publon/10.1111/irv.12910.

## Data Availability

The data that support the findings of this study are available on request from the corresponding author. The data are not publicly available due to privacy or ethical restrictions.
